# Physico-chemical modifications of conjugated linoleic acid for ruminal protection and oxidative stability

**DOI:** 10.1186/1743-7075-5-16

**Published:** 2008-06-01

**Authors:** Hyun-Seuk Moon, Hong-Gu Lee, Chung-Soo Chung, Yun-Jaie Choi, Chong-Su Cho

**Affiliations:** 1 Laboratory of Molecular Signaling, National Institute on Alcohol Abuse and Alcoholism, National Institutes of Health, Bethesda, Maryland 20892-9410, USA; 2 School of Bio-Resources and PNU-Special Animal Biotechnology Center, Pusan National University, Miryang, 627-706, South Korea; 3 College of Agriculture, Life & Environment Sciences, Chungbuk National University, Cheongju 361-763, South Korea; 4 School of Agricultural Biotechnology, Seoul National University, Seoul 151-921, South Korea

## Abstract

Conjugated linoleic acid (CLA) is a mixture of positional and geometric isomers of octadecadienoic acid [linoleic acid (LA), 18:2n-6]. Although ruminant milk and meat products represent the largest natural source of CLA and therefore, their concentration in ruminant lipids are of interest to human health, chemical or physical modifications of CLA should be needed as a means to enhance oxidative stability, to improve post-ruminal bioavailability, and to increase the clinical application. In fact, CLA are rapidly decomposed to form furan fatty acids when its are oxidized in air, and the effectiveness of dietary supplements of CLA may be related to the extent that their metabolisms by rumen bacteria are avoided. For these reasons, many scientists have examined the effect of manufacturing and protection on the stability of CLA in ruminants and food products. In this review, physico-chemical modifications of CLA for ruminal protection such as calcium salt (Ca), formaldehyde protection (FP), lipid encapsulation (LE), and amide linkage (AL), and for oxidative stability such as green tea catechin (GTC), cyclodextrin (CD), arginine (Arg), amylase, and PEGylation are proposed.

## 1. Background

Conjugated linoleic acid (CLA) are a collective term for a group of positional (C8,C10; C9,C11; C10,C12; and C11,C13) and geometric (cis,cis; cis,trans; trans,cis; and trans,trans) isomers of octadecadienoic acid (linoleic acid, LA) with a conjugated double-bond system [1]. Also, the major and most important formation of CLA are the endogenous desaturation of vaccenic acid (VA) due to the action of stearoyl-CoA desaturase (SCD; also so-called delta-9 desaturase) [1,2]. The CLA are effective in protecting tissues from carcinogenesis [2], reducing the development of atherosclerosis [3], stimulating the immune system [4], and inducing enzyme change in mouse liver [5,6]. These effects appear to be mediated by two isomers of CLA, and the two biologically active isomers are the cis9, trans11 and trans10, cis12 [7,8]. Also, it is well known that the CLA content in ruminant-derived food are certainly more affected by the animal diet and production system than by food-manufacturing factors [9]. Several factors influence the CLA content of food products, such as temperature, protein, quality, choice of starter cultures, and period of aging [10]. Variations of CLA content in foods are also affected by the animal's diet, animal age and breed, and seasonal factors [10-12]. Unfortunately, physical or chemical modification of CLA should be needed when its are to be used in food systems as fortifiers or additives. In fact, a number of methods have been used to prepare "rumen-protected" feed supplements, and their efficacy can be described by the extent of protection from rumen bacteria as well as post-ruminal bioavailability [13]. Also, CLA are extremely unstable in air and cis, cis-CLA isomers are most susceptible to oxidative degradation while the four trans, trans-CLA isomers are most stable in air [14], indicating that the CLA must be protected from oxidation. This review is attempted to propose various physico-chemical modification methods of CLA for ruminal protection and oxidative stability, and the potential of clinical applications of CLA is also explained.

## 2. Origin and structure of CLA

### 2. 1. Origin of CLA

Polyunsaturated fatty acids (PUFA) other than CLA, with a conjugated double bond system and with more than two double bonds, occurred naturally in nature in various seed oils but their biological activity had to date not been extensively investigated when compared with data available for CLA [15-18]. The first step in the biohydrogenation of dietary LA resulted in the formation of the cis9, trans11 isomer, due to isomerization and transposition of the delta-12 double bond [16]. This was the most abundant natural isomer present in ruminant tissue fats (over 90% of total CLA) and had been termed rumenic acid (RA) [16,19]. Further hydrogenation of RA resulted in the production of trans11-18:1 VA which was the major trans-monounsaturated fatty acid present in the fats of ruminant food products (milk, yoghurt, cheese, butter, and meats) [19,20]. This contrasts with commercial preparations of CLA where proportions of the two main isomers were usually equal, although the chemical method for synthesis will allow a variety of ratios for the two isomers in the final mixture [16]. Meat from ruminant animals, particularly the fat associated with meat, was also an important source of CLA, contributing in the region of 25 to 30% of the total food intake in Western populations [16,19]. Isomers of CLA could also be synthesized in the laboratory from C18:2 or from sources high in C18:2, such as sunflower, safflower, soybean, or corn oils by a reaction involving alkaline water isomerization [19-22] and isomerization in propylene glycol [20,21,23]. The predominant isomer in milk and other dairy products was the cis9, trans11 with minor but significant proportions of trans10, cis12 [16,19,20].

### 2. 2. Structure of CLA

CLA were a series of positional and geometric isomers of LA where one or both of the double bonds are either in the cis or the trans configuration and transposed to different positions along the acyl chain with the bonds separated by a simple carbon-carbon linkage rather than by the normal methylene group [16,17]. A number of cis-cis, cis-trans, trans-cis, and trans-trans isomers with the double bonds at various locations along the acyl chain, from carbon-6 to carbon-15, had been identified by various chemical reductive, chromatographic, and spectroscopic techniques [16,18,19]. A total of natural CLA isomers had been found in milk, dairy products, beef, human milk, and human adipose tissue using silver ion-high performance liquid chromatography and gas chromatography-electron ionization mass spectrometry [23-26].

## 3. Physiological and biological effects of CLA on health and disease

There have been few studies that have examined the effects of CLA or its isomers in humans. Recently, a wealth of literature available mainly from cell line and animal studies indicates that CLA and individual isomers (especially, C9,T11 and T10,C12) may have numerous health benefits. One of the first studies in healthy adult women examined the effects of 3 g/day intake of CLA for 64 days on fat-free mass, fat mass and percentage fat mass compared to sunflower oil (SFO) placebo, and the results showed that there were no differences in body composition or any of the parameters examined [27]. However, two studies from Norway in healthy exercising humans (CLA, 1.8 g/day) and in overweight and obese humans (CLA, 1.7, 3.4, 5.1, and 6.8 g/day) for 12 weeks showed that CLA can decrease fat mass without significantly affecting body weight [28,29]. Results of the first study in athletes were encouraging considering that a much lower dose of CLA (1.8 g/day) produced significant results compared to the second study where the authors concluded that a dose of 3.4 g/day of CLA was enough to cause reduction of body fat. Another study in 2001 evaluated the effects of intake of 4.2 g CLA/day for 4 weeks on changes in adipose tissue and cardiovascular risk factors in middle-aged obese men with signs of metabolic syndrome [30]. In an interesting study from Netherlands, 13-week intervention with 1.8 or 3.6 g/day CLA in overweight humans supplemented prior to this with a very-low-calorie diet (which induced weight loss) increased resting metabolic rate and lean mass without affecting body weight regain [31]. Other studies had previously showed a net decrease in body fat greater than net decrease in body weight, suggesting that lean mass may have increased in the subjects [28,29]. Volunteers in both these studies were either on intensive training programs or exercised for 90 min, three times per week. These studies seem to suggest that exercise could enhance the fat-lowering effects of CLA and also help improve lean mass in humans. However, there is very limited literature on human studies with individual and different ratios of isomers, which makes it difficult to clearly establish the protective role of the physiological and biologically effect of CLA isomers in improving human health. In fact, recent studies in animals and humans suggest that CLA may not have adverse effects with long-term intervention and may actually be beneficial in reducing fat mass and atherogenic lipids [32]. Also, very few clinical studies have focused on the effects of CLA on bone health and cancer, and there are also differences in the way CLA was supplemented. In addition, effect of CLA and its isomers on inflammatory mediators has not been the subject of extensive research in humans and need to be pursued urgently. Overall, more well-controlled studies are needed before CLA or enriched isomers can be recommended to humans with confidence to improve health and quality of life. Also, evidence for efficacy in humans is being steadily strengthened by the results from clinical trials as well as animal toxicology tests.

## 4. Ruminal protection of CLA

Since ruminant milk and meat products represented the largest natural source of CLA and therefore, their concentration in ruminant lipids was of interest to human health although only trans10, cis12 was primarily mentioned which was used in the studies to obtain milk fat depression [33]. A technology to reduce milk fat output in a controlled manner had a potential application as a management tool [34]. However, the effectiveness of dietary supplements of CLA may be related to the extent that their metabolism by rumen bacteria was avoided [13,34]. Hence, a number of processes had been used to manufacture "rumen-protected" feed supplements, and their efficacies were described by the extent of protection from rumen bacteria as well as post-ruminal bioavailability [13,35]. Several methods had been used to reduce the extent to which lipid supplements were metabolized by rumen bacteria. These included the formation of calcium salts [15,36-38], amide linkages [13,36,37], [39-41], formaldehyde treatment [15], and lipid encapsulation [13,30,42,43]. The form of fatty acid required for the production of these supplements varies, and became an important consideration due to the variation in manufacturing processes and costs associated with the production of different lipid forms.

### 4. 1. Calcium salt

**Figure 1:**
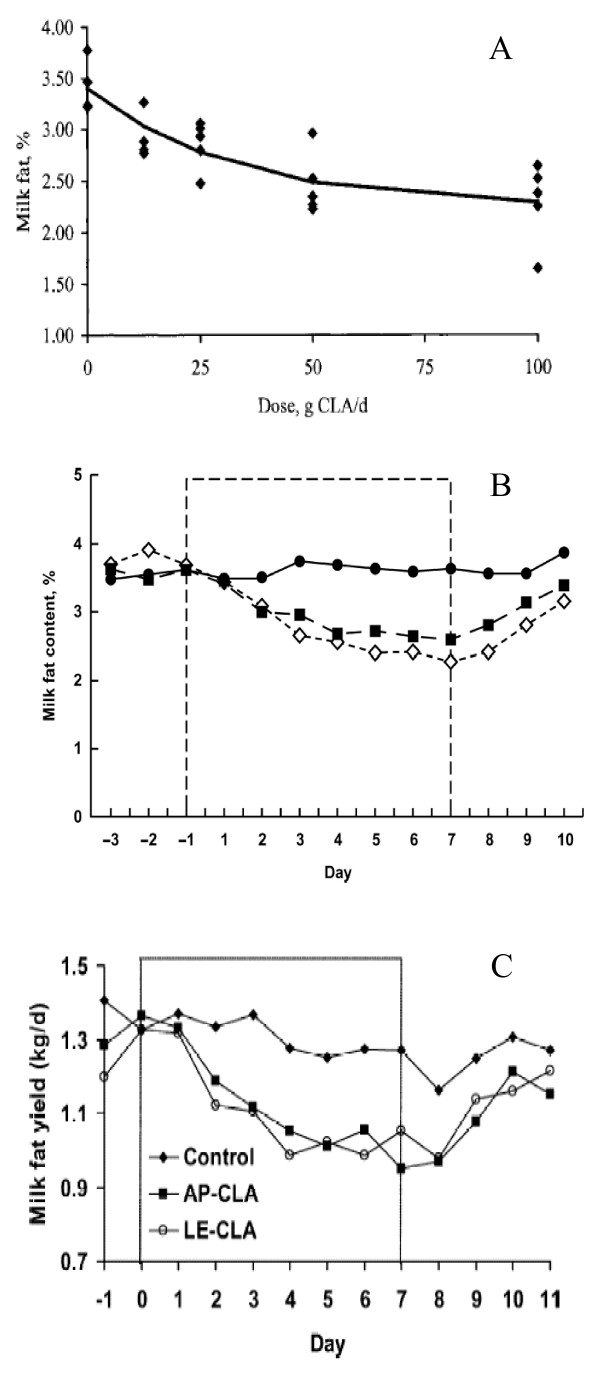
**(A) **Relationship between dose of conjugated linoleic acid (CLA) as calcium salts and milk fat percentage in Holstein cows on the fifth days of CLA feeding. Graph illustrates predicted exponential regression model (y = 1.114 * e^-0.033x ^+ 2.249) (*J Dairy Sci *2002, 85:2023–2029). **(B) **Temporal pattern of milk fat content in cow (n = 3) during intraruminal infusion of different rumen-protected conjugated linoleic acid (CLA) formulations. Treatments were control (no added lipid; •), calcium salt of CLA (▪), and formaldehyde-protected CLA (◇) (*J Dairy Sci *2005, 88:1685–1693). **(C) **Temporal pattern of milk fat yield during supplementation of rumen-protected CLA supplements [amide-protected CLA (AP-CLA): lipid-encapsulated-CLA (LE-CLA)] (*J Dairy Sci *2004, 87:3010–3016)

Calcium salts (Ca) of fatty acids had been commercially used as a dietary lipid supplement for dairy cows, and they had been experimentally used to provide rumen-protected supplements of CLA [15,36]. Giesy et al. reported that milk fat percentage was reduced in a dose-dependent manner by feeding Ca-CLA [36]. The predicted equation followed data well and showed the expected decrease in milk fat percentage as dose increased, indicating that Ca-CLA provided with the opportunity to regulate milk fat synthesis with only a minor dietary addition (Fig. 1A). These effects were caused by the trans10, cis12 CLA. The extent of the effect was certainly higher by using the form of a Ca because it protected the fatty acid from microbial degradation. Moreover, supplementing dairy cows with rumen-protected forms of CLA such as Ca-CLA substantially reduced the yield and content of milk fat without altering other production responses [15]. Similar effects on milk fatty acid pattern had been reported in previous studies where cows had received Ca-CLA [37], and studies involving abomasal infusion of trans10, cis12 CLA, where effects on *de novo *synthesized fatty acids became more pronounced as the dose of trans10, cis12 CLA increased [38].

### 4. 2. Formaldehyde protection

Formaldehyde protection (FP) enabled the use of either FFA or esterified fatty acids [15]. Figure 1B and Table 1 showed that the CLA supplements resulted in a progressive reduction in milk fat content through the first few days of the treatment period and the proportion of preformed fatty acids in milk fat was increased with CLA treatment, whereas short- and medium-chain fatty acids tended to decrease, indicating that both protection forms such as Ca and FP were effective methods for the formulation of CLA supplements to induce milk fat depression (MFD) in lactating dairy cows and the transfer of trans10, cis12 CLA into milk fat for both Ca-CLA and FP-CLA supplements was much lower than that previously reported when CLA were supplied post-ruminally. As expected, the CLA treatments resulted in increased concentrations of trans10, cis12 CLA in milk fat, with the increase for FP-CLA treatment being greater than the Ca-CLA treatment.

**Table 1:** Intake and milk production results during intraruminal administration of rumen-protected conjugated linoleic acid (CLA) (*J Dairy Sci *2005, 88:1685–1693).

	**Treatment**^1^				***P***	
			
	**Control**	**Ca-CLA**	**FP-CLA**	**SEM**	**CLA**^2^	**Type**^3^
DMI, kg/d	23.6	23.1	23.3	1.13	0.68	0.84
Milk yield, kg/d	21.9	20.6	19.5	2.32	0.31	0.57
Milk fat						
%	3.61	2.61	2.34	0.27	< 0.01	0.18
g/d	788	517	441	42	0.02	0.30
Milk protein						
%	3.16	3.38	3.48	0.17	0.08	0.40
g/d	690	673	669	50	0.64	0.93
SCC, ×1000	150	114	137	98	0.44	0.52

^1^Values represent mean from d 6 and 7 for control (no added lipid) and intraruminal administration calcium salts of CLA (Ca-CLA) and formaldehyde-protected CLA (FP-CLA).

^2^Contrast between control and the combined CLA treatments.

^3^Contrast between Ca-CLA and FP-CLA treatments.

### 4. 3. Lipid encapsulation

Recent work showed that the FFA and methyl ester forms of trans10, cis12 CLA were equally effective in reducing milk fat synthesis when supplied by abomasal infusion [39]. Also, Perfield et al. observed a gradual reduction in milk fat yield over the first few days of treatment and a return to control values when the supplement was terminated [13], and this was similar to changes observed when trans10, cis12 CLA were abomasally infused [42] or provided intravenously [43]. It was found that the lipid encapsulation (LE) of CLA supplement was manufactured by binding methyl esters of CLA to a silica matrix, and then coating this complex with hydrogenated soybean oil, which contained fatty acids in the triglyceride form.

### 4. 4. Amide linkage

Although Ca and FP of fatty acids had been commercially used as a dietary lipid supplement for dairy cows, and they had also been experimentally used to provide rumen-protected supplements of CLA [36,37], only few studies had been performed to suggest the rumen-protected supplements of CLA on amide protection (AP) methods. A simple AP supplement required that the starting material be FFA, whereas other amide-protected supplements had been manufactured from oils, and esters or other forms could be used for FP or LE [40]. Perfield et al. examined the use of AP and LE as methods to supply CLA [13]. It was found that the AP supplement provided CLA as FFAs, whereas the LE supplement provided CLA as methyl esters, suggesting that the AP-CLAs supplements resulted in decreased secretion of milk fatty acids of all chain lengths, but the reduction was relatively greater for milk fatty acids containing ≤ 16 carbons (Fig. 1C and Table 2). These results indicated that the AP-CLA supplements were able to reduce milk fat in a controlled manner with no adverse effects. Recent work had also reported that the FFA and methyl ester (ME) forms of trans10, cis12 CLA were equally effective in reducing milk fat synthesis when supplied by abomasal infusion [39] and CLA-ME could be protected from ruminal metabolism and inclusion of RP-ME-CLA supplement in the diet reduced milk fat content by 35–40% and significantly increased the concentration of CLA isomers in milk [41]. Overall, supplementation of an AP-CLA or a LE-CLA product resulted in similar reductions in milk fat with no effect on feed intake or milk yield. Although reduction in milk fat yield had achieved nadir by the sixth day of supplementation, transfer of trans10, cis12 CLA into milk fat was similar for both rumen-protected CLA products, and the AP-CLA and LE-CLA supplements were able to reduce milk fat in a controlled manner with no adverse effects [13], as this was a short-term study with very limited animal numbers, further research with these supplements would be needed to verify and extend results.

**Table 2:** Performance of lactating dairy cows receiving rumen-protected supplements of conjugated linoleic acid (CLA)^1,2 ^(*J Dairy Sci *2004, 87:3010–3016).

	**Treatment**				
			
**Variable**	**Control**	**AP-CLA**	**LE-CLA**	**SEM**	***P***^3^
DMI, kg/d	30.6	31.6	30.4	0.9	0.50
Milk, kg/d	40.5	42.6	42.7	3.5	0.32
Milk fat					
%	3.23^a^	2.37^b^	2.34^b^	0.15	< 0.001
kg/d	1.272	1.004^b^	0.992^b^	0.079	< 0.001
Milk protein					
%	2.55	2.51	2.58	0.12	0.07
kg/d	1.001^b^	1.063^a^	1.086	0.023	0.02

^a,b^Means within a row with different superscripts differ.

^1^Supplements were amide-protected CLA (AP-CLA) and lipid-encapsulated CLA (LE-CLA).

^2^Values represent an average of day 6 and 7 of supplementation.

^3^Statistical probability of treatment effects.

## 5. Oxidative stability of CLA

Some of the studies had shown that CLA acted as an anti-oxidant although some other studies had been reported that CLA might be pro-oxidant. Anti-oxidant activity of CLA was observed in mammary gland tissues from rats fed with CLA, when evaluated by the thiobarbituric acid reactive substances (TBARS) values [44]. However, CLA did not reduce TBARS values in pork patties mixed with CLA [44,45] and in *in vitro *study; CLA were oxidized as rapidly as LA [12,46]. In fact, CLA were extremely unstable in air and cis, cis-CLA isomers were most susceptible to oxidative degradation while the four trans, trans-CLA isomers were most stable in air under same conditions [14]. Moreover, CLA were oxidized faster than LA, suggesting that a conjugated double bond was more vulnerable to auto-oxidation than a non-conjugated double bond. This was in agreement with those of previous observations [14,47,48], indicating that CLA must be protected from oxidation when it was to be used in food systems as fortifiers or additives.

### 5. 1. Green tea catechins

**Figure 2:**
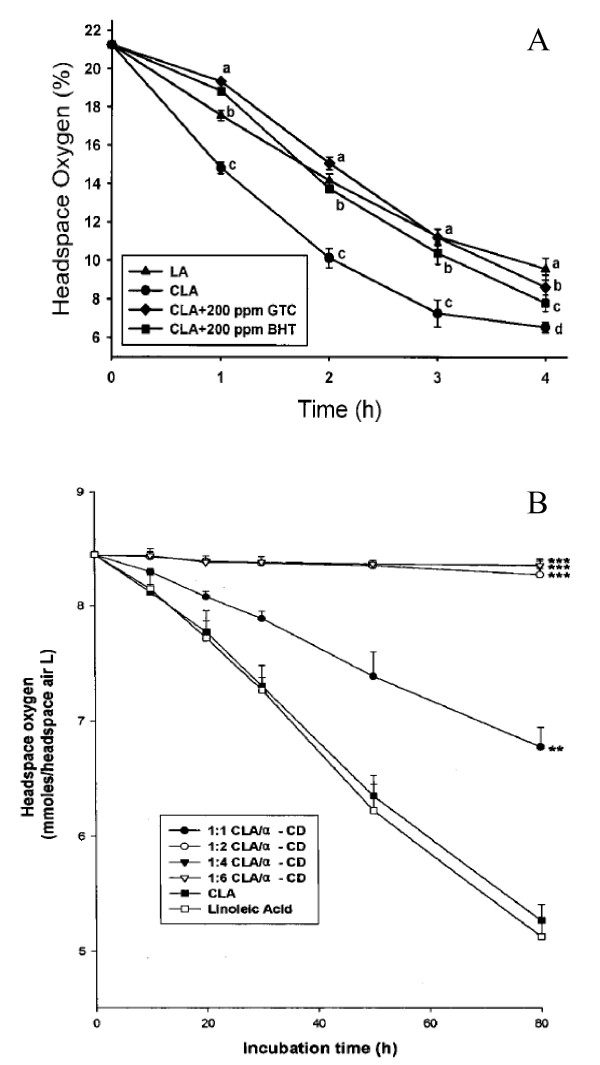
**(A) **Headspace-oxygen consumption profile of LA and CLA with or without addition of 200 ppm GTC or 200 ppm BHT at 90°C. Data are expressed means ± SD for n = 5 samples. Means at the same time point with different superscript letters (a-d) differ significantly (p < 0.05) (*J Agric Food Chem *2000, 48:3072–3076). **(B) **Headspace-oxygen depletion by CLA/α-CD microencapsulations. The CLA/α-CD microencapsules were incubated in a shaking incubator (35°C, 250 rpm) for a period 80 h. Two and three asterisks represent that headspace-oxygen depletion is significantly different from that of the control, incubated for 80 h, at p < 0.01 and p < 0.001 levels, respectively, by Tukey's w significant test (*J Agri Food Chem *2000, 48:3922–3929).

Dietary supplements containing green tea extracts were expected to be comprised of polyphenolic compounds called catechins, which were commonly obtained from green tea. The catechins were believed to act as anti-oxidants and free radical scavengers having chemo-preventative behavior as well as protection against coronary heart disease and attenuation of high blood pressure [40]. The catechins common to all green teas were (-)-epigallocatechin (EGC), (+)-catechin (C), (-)-epigallocatechin-3-gallate (EGCG), (-)-epicatechin (EC), and (-)-epicatechin gallate (ECG) [49]. Also, it was known that green tea catechins were strong anti-oxidants [50]. Seo et al. reported that CLA was more stable than LA in the aqueous system when 2,2'-azobis(2-amidinopropan) dihydrochloride was used as a free radical initiator [51]. Also, jasmine green tea catechins (GTC) (200 ppm) were effective as an anti-oxidant in protecting CLA from oxidation and the inhibition of 200 ppm GTC on CLA oxidation was even stronger than that of 200 ppm butylated hydroxytoluene (BHT) under the same conditions [40]. The oxygen consumption test showed that the oxygen uptake by the CLA samples was considerably more unstable than LA, whereas addition of 200 ppm GTC significantly decreased the oxygen uptake by CLA as compared with the control CLA samples (Fig. 2A). It is noteworthy that 200 ppm GTC is more effective than 200 ppm BHT in protecting CLA from oxidation. It is known that canola oil contains α-tocopherol and it is also possible that GTC and α-tocopherol have a synergistic effect on the oxidation of CLA when added in canola oil [52]. Also, feeding canola seed to lactating dairy cows resulted in milk fat with higher proportions of healthful fatty acids without affecting milk yield or composition of milk [53].

### 5. 2. Cyclodextrin/CLA complex

α-, β-, and γ-cyclodextrins, consisting of 6, 7, and 8 D(+)-glucopyranose units connected by α(1→4) glucosidic bond, respectively, are well known cyclodextrins (CDs) for food industry [54]. The CDs were known to form inclusion complexes with various compounds, ranging from polar to highly non-polar agents [55]. Also, PUFA encapsulated in α- and β-CDs had been shown to protect completely against oxidation even in pure oxygen [56]. Oxidative stability of CLA encapsulated in α-, β-, and γ-cyclodextrins (designated as CLA/CDs microencapsules) was studied by measuring the headspace-oxygen depletion in airtight serum bottles and by measuring the peroxide values (POV) [57]. It was found that CLA/α-CD microencapsules at a 1:4 mole ratio completely protected CLA from oxidation, when oxidized at 35°C and a 1:6 mole ratio of CLA/β-CD was required to give a protective effect similar to that exhibited by CLA/γ-CD microencapsules at a 1:4 mole ratio (Fig. 2B). The protective efficiency of CDs for CLA oxidation may be, in part, attributed to the hydrophobicity of the inner cavity of CDs, which facilitated insertion of the conjugated diene portion into the CD cavity, and the cavity of the CDs, which was possibly large enough to incorporate some oxygen molecules and create a miniature reaction chamber to facilitate the reaction between CLA and oxygen, suggesting that physical interference by CDs was not a negligible factor for the oxidative stability of CLA. Further study will be required to explore the structural features of CLA/CDs microencapsules. It was of significance to note that β-CD was an appropriate material with which to microencapsulate CLA for industrial use, because of its adequate protective effects for CLA oxidation and because it was much lower in cost than other CDs.

### 5. 3. Arginine/CLA complex

**Figure 3:**
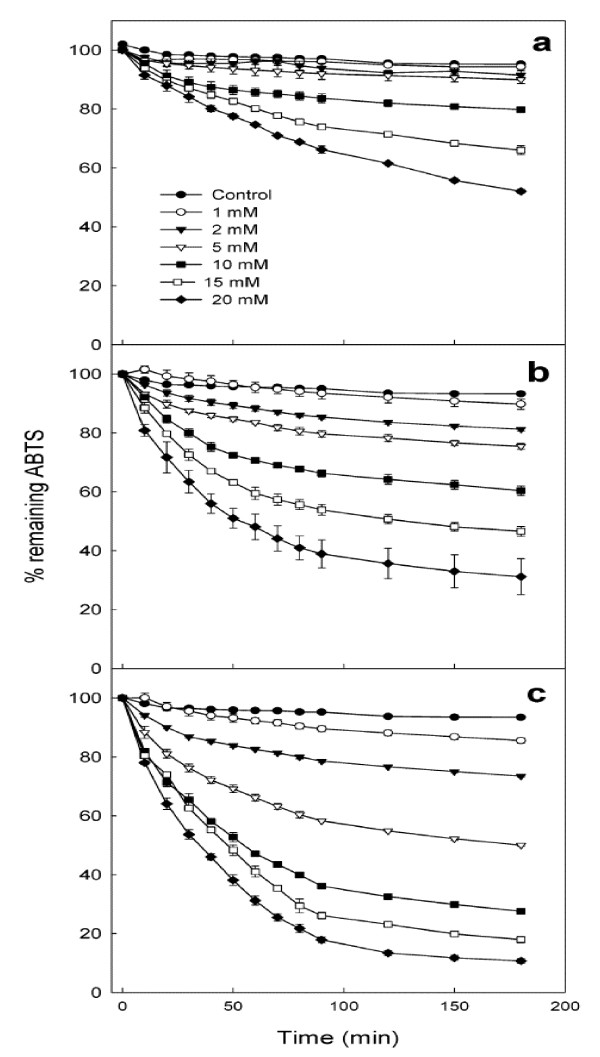
Kinetics of reaction of ABTS radicals with (a) CLA, (b) Arg, and (c) Arg-CLA. Each test compound at 0, 1, 2, 5, 10, 15, and 20 mM was reacted with 100 μM ABTS radicals. Error bars represent standard deviations of each data point (n = 3) (*J Agric Food Chem *2004, 52:439–444).

Arginine (Arg), a water-soluble amino acid, was known to be a substrate of nitric oxide synthase and regulate vascular function and blood pressure homeostasis, and thus prevent cardiovascular disease [58]. Also, Arg had some protective roles against oxygen radical attack possibly due to its direct chemical interaction with oxygen radicals [59]. Kim et al. reported that hydroperoxide production from CLA increased about 12-fold at 10 hr after heat treatment at 100°C, whereas Arg/CLA complex did not exhibit significant hydroperoxide production [60]. The Arg/CLA complex showed synergistic anti-oxidant activity in a 2,2'-azinobis(3-ethylbenzothiazoline)-6-sulfonic acid (ABTS) radical scavenging assay (Fig. 3). It was found that Arg/CLA complex at 20 mM scavenged 89% of ABTS radicals in 3 h, whereas CLA alone quenched only 48% under the same conditions, suggesting that a hydrophilic Arg/CLA complex exhibited enhanced oxidative stability and anti-oxidant activity, which may expand the scope of CLA applications in various food industries. Recent research had been aimed to elucidate the physiological background of CLA-inducing reduction in adipose tissue mass [61,62]. However, some concerns had been aroused on the potential implication of the CLA in insulin resistance and fatty liver under certain conditions [63-65]. Because Arg infusion was known to have a preventive role in the insulin resistance by decreasing the total plasma homocysteine concentration [66] and antioxidant capacity [66,67], the formation of Arg/CLA complex could alleviate the potential side effects, if any, resulted from the high dose of CLA. Thus, the presence of Arg in the form of complex with CLA may expand the scope of the application of CLA as a health-promoting agent.

### 5. 4. Amylase/CLA complex

**Figure 4:**
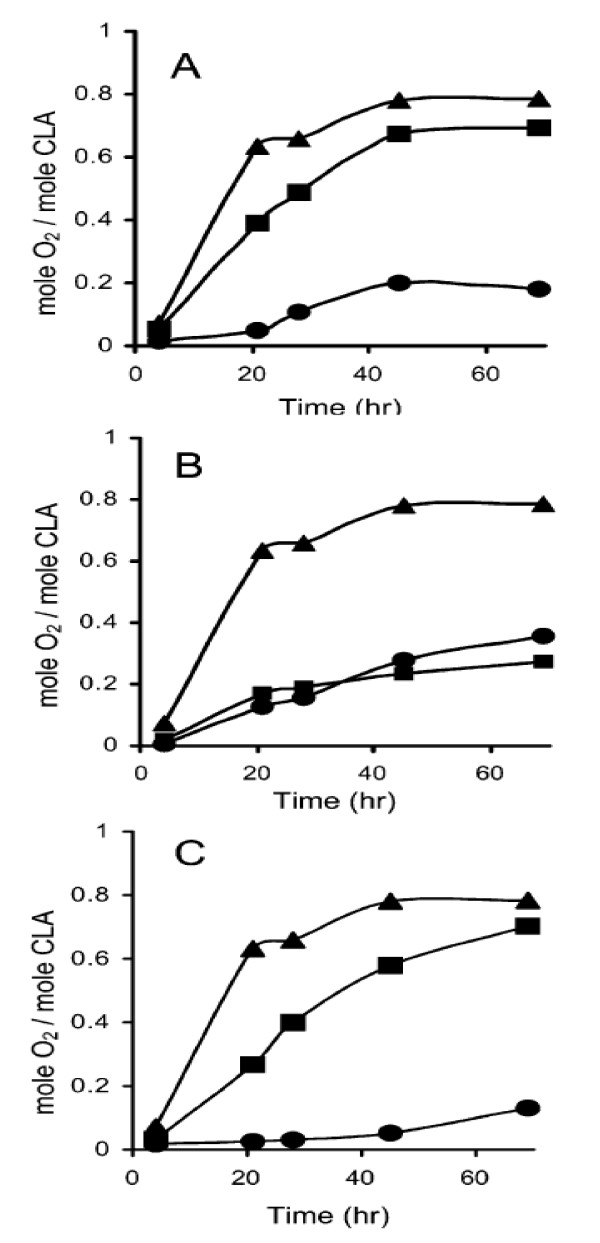
Headspace-oxygen analysis of complexes created at 90°C (A), 60°C (B), and 30°C (C) in water/DMSO solution (•) and by KOH/HCl solution (▪), with comparison to free CLA(▲) (*Biomacromolecules *2005, 6:121–130).

Most of the interest in amylase/lipid complexes focused on their technological importance in the starch food system, since they modified the texture and structural stability of starch based-product (e. g., reduction in stickiness, improved freeze-thaw stability, and retardation of retrogradation) [68,69]. Other researchers studied amylase/lipid complexes in view of their contribution to the bioavailability of starch, in terms of its enzymatic digestion [70]. It was shown that the V-form could be produced from mono- and di-glycerides, and saturated fatty acids, as well as un-saturated fatty acids [70,71]. The protection against oxidation afforded to CLA by its inclusion in an amylase complex demonstrated the potential of the complexes, especially those created in water/dimethyl sulfoxide (DMSO) solution, to efficiently protect CLA from oxidation [72]. It was shown that the peaks of the diffractograms obtained from complexes made in KOH/HCl solution were narrower than those obtained from complexes created by water/DMSO solution, indicating that these complexes were composed of large crystals. Also, the complexes created in water/DMSO solution at 90°C revealed globural structures of heterogeneous nature with an average z-range of 71.6 ± 59 nm and diameter of 152 ± 39 nm. Fig. 4 shows oxidation of CLA on each water/DMSO and KOH/HCl solution, indicating that the protective effect of complexes against CLA oxidation was higher for complexes created in water/DMSO solution than that for KOH/HCl solution, suggesting that complexes created in water/DMSO solution exhibited better protective ability from oxidation. Hence, these results indicate that the amylase/lipid complex system could serve as a vehicle for delivery of PUFA to the intestine and potential use of amylase/lipid complexes could be supplementation of various stable foods with PUFA.

### 5. 5. PEGylation

**Figure 5:**
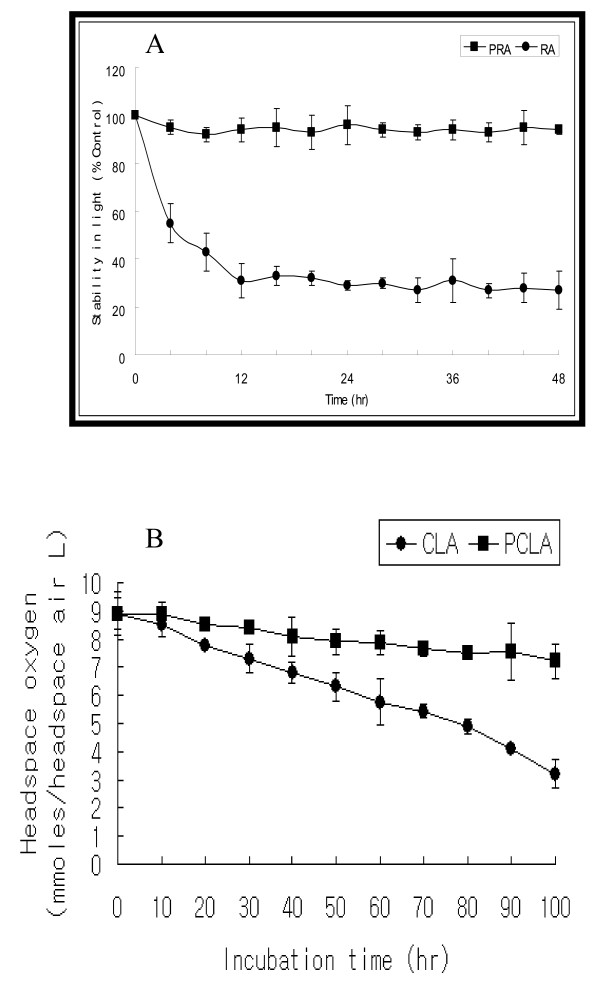
**(A) **Stability test of PRA. At specific time intervals, changes in the absorbance were determined through UV absorbance spectroscopy at 350 nm. The concentration of intact RA in the methanol solution rapidly decreased as compared to that of PRA during incubation at room temperature under light exposure. Each experiment was performed in triplicate. Values are means ± SD (*J Nutr Biochem *2007, 18:322–331). **(B) **Headspace-oxygen depletion by CLA and PCLA. Headspace oxygen in the sample bottles was analyzed by injecting a 100 μL headspace air sample into a HP 5890 GC (Avondale, PA), equipped with a stainless steel molecular sieve column (13×, 80:100; Alltech, Deerfield, IL) and a thermal conductivity detector. High purity helium (99.99%) was used as the carrier gas. The flow rate was 40 mL/min. The GC oven temperature was maintained at 40°C. The injector port and detector temperatures were maintained at 120 and 150°C, respectively. Each sample was analyzed in triplicate. Oxygen contents were quantified by an HP 3396A integrator (unpublished data).

Poly(ethylene glycol) (PEG) was a nontoxic, water-soluble polymer widely used for stabilizing colloids in foods and paints and in formulating pharmaceuticals and cosmetics [73]. Generally, covalent attachment of activated PEG to proteins altered protein properties, such as increased solubility and stability in organic solvents, increased thermal stability, and reduced immunogenicity and antigenicity, in ways that extend their potential uses [74-76]. Also, PEGylation provided a higher stability owing to the formation of core-shell type nanoparticels (NPs) when compared to the non-modified drug [77]. In fact, PEGylated drugs such as core-shell type polymeric NPs increased stability in air, light, pH, and temperature [74,76]. Support for these results, as shown in Fig. 5A, the concentration of intact *all-trans *retinoic acid (atRA) in the methanol solution rapidly decreased during incubation at room temperature under light exposure whereas the rate of PEGylated atRA (PRA) degradation was very slow [78]. Also, we previously reported that PEGylated CLA (PCLA) had increased bioavailability than CLA due to the biocompatible and hydrophilic properties of PEG, and peroxisome proliferator activated receptor gamma 2-induced adipogenesis was reduced by PCLA [79]. In addition, we further demonstrated that a time-dependent effect on lipolysis and p-extracellular signal-related kinases (ERK) expression was observed for PCLA-treated, but not for CLA-treated cultures [80], suggesting that the induction by PCLA of mitogen-activated protein kinase kinase (MEK)/ERK mitogen-activated protein kinase (MAPK) activation was linked to secretion of adipo-cytokines, interleukin-6 (IL-6), and interleukin-8 (IL-8), in time-dependent manners. Our findings provide support for a role for PCLA as a pro-drug in the regulation of metabolism in adipose tissue. Moreover, we showed that the level of headspace oxygen decreased by PCLA was lower than that decreased by CLA, indicating that the oxidation of CLA was significantly protected by PEGylation, as shown in Fig. 5B (unpublished data). Hence, observations with PEGylation regarding their biological and CLA-protected effects are encouraging and their use in a variety of biological activity, oxidative stability and post-ruminal bioavailability must not be ignored.

## 6. Summary

CLA have numerous potential health benefits, and the fats from milk and meats of ruminants are the richest natural dietary sources of CLA. Manipulating the diets of dairy and beef cattle and altering management practices on the farm could enhance the CLA contents of milk and dairy products and beef products. Also, the CLA contents of milk, dairy products, meat, and meat products vary widely, and the CLA intake by humans have the potential to increase to a level that have been shown to reduce the incidence of cancer in animal models through the consumption of CLA-enriched dairy and beef products. However, CLA are rapidly decomposed to form furan fatty acids when its are oxidized in air, and the effectiveness of dietary supplements of CLA may be related to the extent that their metabolism by rumen bacteria is avoided. To overcome with these phenomena, many investigations have examined the effect of manufacturing and protection on stability of CLA in ruminants and food products such as rumen-protected methods including Ca, Fa, AP, and LE, and oxidative stability methods such as GTC, CD, Arg, and amylase. All of these modifications, however, there are short-term study with very limited animal numbers; further research with these supplements will be needed to verify and extend results. Also, before CLA supplementation are recommended for human beings, controlled research studies using single isomers of CLA need to be completed to determine its efficacy and safety. Moreover, it is still questionable whether high levels of trans10, cis12 in milk are indeed desired-especially taking into consideration that trans10, cis12 is not really a naturally occurring fatty acid although CLA, specifically trans10, cis12, might be detrimental to human health. However, suffice it to say that observations with CLA regarding their possible health effects are encouraging and their use in a variety of functional foods are a distinct possibility that should be interested.

## Abbreviations

CLA: Conjugated linoleic acid; DHA: docosahexanenoic acid; ACA: arachidonic acid; LA: linoleic acid; PUFA: polyunsaturated fatty acid; FFA: free fatty acids; Ca-CLA: calcium salts-CLA; FP-CLA: formaldehyde-protected-CLA; MFD: milk fat depression; AP: amide protection; LE: lipid encapsulation; CLA-ME: CLA-methyl esters; TBARS: thiobarbituric acid reactive substances; EGC: (-)-epigallocatechin; C: (+)-catechin; EGCG: (-)-epigallocatechin-3-gallate; EC: (-)-epicatechin; ECG: (-)-epicatechin gallate; GTC: green tea catechins; BHT: butylated hydroxytoluene; CDs: cyclodextrins; POV: peroxide values; Arg: Arginine; ABTS: 2,2'-azinobis(3-ethylbenzothiazoline)-6-sulfonic acid; DMSO: dimethyl sulfoxide; PEG: Poly(ethylene glycol); BSA: bovine serum albumin; β-lg: β-lactoglobulin; PCLA: PEGylated CLA; NPs: nanoparticles; RA: rumenic acid; VA: vaccenic acid.

## 7. Authors' contributions

MHS designed and wrote this manuscript. LHG, CCS, CYJ, and CCS conceived of the study, and participated in its design and co-ordination. All authors read and approved the final manuscript.
